# Paralog-dependent isogenic cell assay cascade generates highly selective SLC16A3 inhibitors

**DOI:** 10.1016/j.chembiol.2023.06.029

**Published:** 2023-08-17

**Authors:** Vojtech Dvorak, Andrea Casiraghi, Claire Colas, Anna Koren, Tatjana Tomek, Fabian Offensperger, Andrea Rukavina, Gary Tin, Elisa Hahn, Sarah Dobner, Fabian Frommelt, Andras Boeszoermenyi, Viktoriia Bernada, J. Thomas Hannich, Gerhard F. Ecker, Georg E. Winter, Stefan Kubicek, Giulio Superti-Furga

**Affiliations:** 1 CeMM Research Center for Molecular Medicine of the Austrian Academy of Sciences, 1090 Vienna, Austria; 2 Department of Pharmaceutical Sciences, University of Vienna, 1090 Vienna, Austria; 3 Center for Physiology and Pharmacology, Medical University of Vienna, 1090 Vienna, Austria

**Keywords:** Solute Carrier Transporters, SLC, Chemical Screening, Cell-based Assay, SLC16, SLC16A3, MCT4, Chemical Probe, Chemical Proteomics

## Abstract

Despite being considered druggable and attractive therapeutic targets, most of the solute carrier (SLC) membrane transporters remain pharmacologically underexploited. One of the reasons for this is a lack of reliable chemical screening assays, made difficult by functional redundancies among SLCs. In this study we leveraged synthetic lethality between the lactate transporters SLC16A1 and SLC16A3 in a screening strategy that we call paralog-dependent isogenic cell assay (PARADISO). The system involves five isogenic cell lines, each dependent on various paralog genes for survival/fitness, arranged in a screening cascade tuned for the identification of SLC16A3 inhibitors. We screened a diversity-oriented library of ∼90,000 compounds and further developed our hits into slCeMM1, a paralog-selective and potent SLC16A3 inhibitor. By implementing chemoproteomics, we showed that slCeMM1 is selective also at the proteome-wide level, thus fulfilling an important criterion for chemical probes. This study represents a framework for the development of specific cell-based drug discovery assays.

## Highlights

• Paralog-dependent isogenic cell assay exploits genetic interactions
• Isogenic cells with individual dependency on SLC16A1, SLC16A3, SLC16A7, and SLC16A8
• Chemical screening uncovering two series of compounds selective to SLC16A3
• Development of SLC16A3 chemical probe

## Graphical abstract

**Figure:**
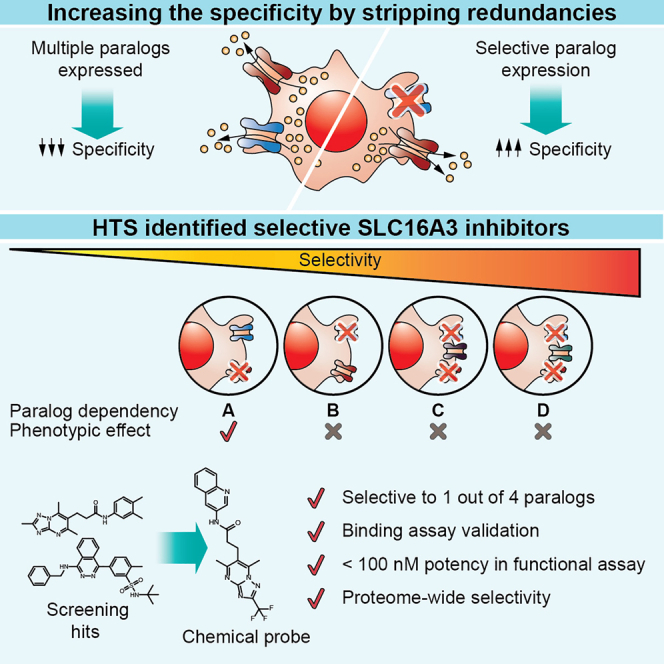

## Teaser

Dvorak et al. report the development of a chemical probe targeting SLC16A3 using an assay system relying on a series of isogenic cell lines with engineered dependency on individual paralogs of SLC16A3. This system overcomes functional redundancies that hamper the development of specific cell-based screening assays.

## Introduction

The solute carrier (SLC) superfamily of membrane transporters plays an important role in cellular homeostasis and metabolism. An increasing number of SLCs is being linked to different human diseases, which is reflected in a growing interest in SLC-oriented drug discovery.^1^^,^^2^ Yet if compared with other protein families, the SLC superfamily remains pharmacologically underexploited.^3^ This originates on one hand from the fact that most SLCs are relatively understudied and on the other hand from a lack of tools, such as chemicals targeting SLC function or appropriate biological assays.^4^

Development of biophysical assays using purified proteins is challenging due to the complexity of SLC structures, containing multiple transmembrane-passing helices and hence complicated protein purification. Development of cell-based assays, on the other hand, is hampered by frequent redundancies in the SLC function, and expression of multiple SLCs capable of transporting same substrates by the same cell, representing a challenge for assay specificity.^1^^,^^5^ Ideally, a chemical screening assay would combine the advantages of the physiological, cellular context of the target with the precision of assays employing purified protein. This could be attempted by engineering a cell-based system with reduced functional genetic redundancies. One possibility how to identify such redundancies is to exploit genetic interactions, in particular synthetic lethality.^6^ This concept was originally derived from fly and yeast genetics and later rationalized for finding cancer specific drug targets. Unbiased genetic interaction screens showed that genetic dependencies are very powerful in uncovering functional overlap and relatedness between genes involved in similar cellular processes.^7^^,^^8^ While the exploitation of genetic interactions for drug discovery has been so far mostly oriented toward synthetic lethality as a vulnerability of cancer cells,^9^ in principle this concept could be leveraged to uncover functional redundancies and to increase the functional resolution of cell-based systems. This could be particularly useful in protein groups like SLCs, where a large portion of the superfamily remains orphan, and thus the identification of functional overlaps is challenging.

Although lactate is sometimes viewed simply as a metabolic waste product, it is becoming clear that it has a role as a circulating nutrient as well as signaling molecule.^10^^,^^11^ In fact, lactate can efficiently shuttle carbon atoms between different tissues, as well as different cell types within tissues, and is a major substrate for the TCA cycle.^12^ Monocarboxylate transporters SLC16A1 (MCT1), SLC16A3 (MCT4), SLC16A7 (MCT2), and SLC16A8 (MCT3) are responsible for the transport of lactate across the plasma membrane and play a critical role in this process.^13^ Even though the functions of individual SLC16 lactate transporters can be considered redundant to some extent, SLC16A1 is typically considered the major lactate importer while SLC16A3 is a major lactate exporter, mainly due to the differences in affinities to lactate and pyruvate.^13^ Similarly, all four paralogs vary in their expression pattern across tissues, as well as cell lines, with, at one extreme, SLC16A1 being expressed ubiquitously, and at the other extreme, SLC16A8 being exclusively expressed in retinal tissue.^14^ Although all four paralogs are highly similar in their structures, the degree of similarity between them varies. While SLC16A1 and SLC16A3 share 39% of amino acid identity, the identity between SLC16A1 and SLC16A7 is 59% and the identity between SLC16A3 and SLC16A8 is 57%.^15^ The structural similarity represents a challenge for development of selective inhibitors. This is illustrated by the existing SLC16A1 inhibitors, such as AZD3965 or BAY-8002, that display decent selectivity over SLC16A3, but lack selectivity over SLC16A7.^16^ Modulation of the levels of lactate through targeting lactate transport was proposed as a target in several therapeutic areas, including cancer, inflammatory diseases, and heart hypertrophy (recently reviewed in^17^). While SLC16A1 inhibitors exist already for many years, and the most clinically advanced SLC16A1 inhibitor recently completed a phase I trial,^18^ inhibitors for SLC16A3 have been emerging only recently.^19^^,^^20^^,^^21^^,^^22^^,^^23^

In this study, we have implemented the synthetic lethality concept to focus a phenotypic screening assay on SLC16A3. By manipulating the expression of lactate transporters in HAP1 cells, we created a paralog-dependent isogenic cell line (PARADISO) assay strategy, employing a series of perfectly isogenic cell lines, individually dependent on four different paralog genes for their fitness/survival, or the original wild-type cell line as a control. PARADISO is arranged in a cascade logic that allowed us to screen a diversity-oriented library of ∼90,000 compounds. Two chemotypes inhibiting SLC16A3 in a highly selective manner were discovered through the PARADISO screen. We have further validated both chemotypes with orthogonal assays and derived slCeMM1, an SLC16A3 inhibitor with potency <100 nM and great selectivity over the other three additional paralogs. Finally, by implementing chemical proteomics, we showed that slCeMM1 is selective also at the proteome-wide level, fulfilling a chemical probe criterion.

## Results

### Development of a paralog specific screening assay

**Figure 1:**
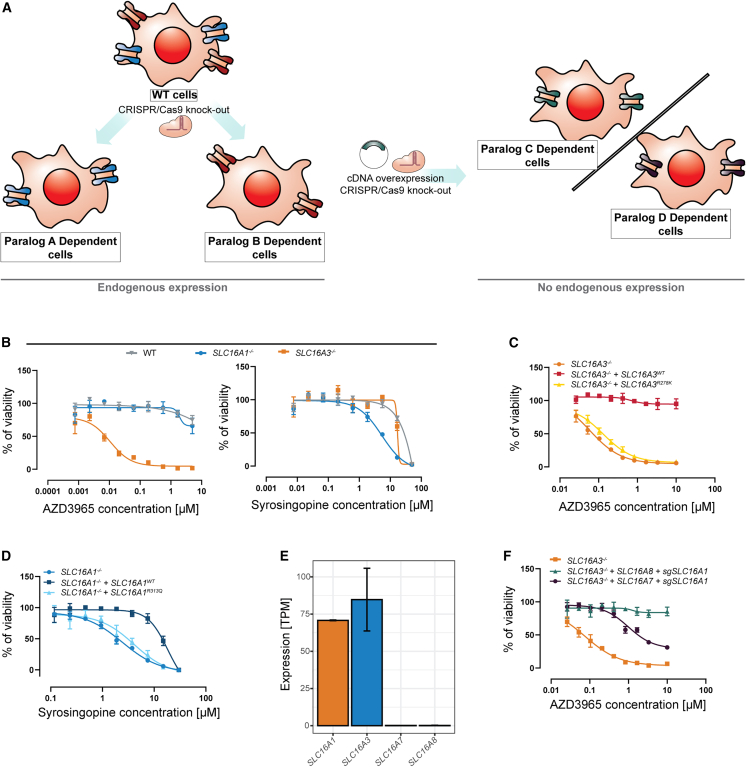
PARADISO assay validation for SLC16 family lactate transporters (A) Paralog-dependence isogenic cell assay logic. (B) Sensitivity of HAP1 WT, HAP1 *SLC16A1*^*−/−*^, and HAP1 *SLC16A3*^*−/−*^ cell lines to AZD3965 (SLC16A1/SLC16A7 inhibitor) and Syrosingopine (SLC16A3/SLC16A1 inhibitor). (C) Sensitivity of HAP1 *SLC16A3*^*−/−*^ and HAP1 *SLC16A3*^*−/−*^ with *SLC16A3*^*WT*^ or *SLC16A3*^*R278K*^ overexpression to AZD3965. (D) Sensitivity of HAP1 *SLC16A1*^*−/−*^ and HAP1 *SLC16A1*^*−/−*^ with *SLC16A1*^*WT*^ or *SLC16A1*^*R313Q*^ overexpression to Syrosingopine. (E) RNA seq data showing expression of lactate transporters in HAP1 WT cells. (F) Sensitivity of SLC16A7 (*SLC16A3*^*−/−*^ + *SLC16A7* + *sgSLC16A1*) and SLC16A8 (*SLC16A3*^*−/−*^ + *SLC16A8* + *sgSLC16A1*) dependent cells to AZD3965. All data are reported as mean ± SD.

To develop a paralog-specific screening system, we started from an SLC genetic interaction network recently reported for the HAP1 haploid human cell line.^24^ This is a very well characterized cell line, for which the genome and proteome have been described, and in which we have been able to identify the so-called essentialome, all genes functionally required for fitness under standard cell culture condition.^25^ By virtue of being haploid and otherwise preserve all salient features and pathways of other tissue culture cell lines, it allows for genomic engineering without interference of a second (or often multiple, such as in HEK293 and HeLa) allele.^26^^,^^27^ As a proof-of-concept we focused on SLC16A1 and SLC16A3, lactate transporters with a strong and reciprocal genetic interaction (Figure S1A). Multiple studies previously noted a functional redundancy between SLC16A1 and SLC16A3 and reported frequent sensitivity to inhibition of SLC16A1 in cell lines not expressing *SLC16A3*.^16^^,^^28^^,^^29^^,^^30^^,^^31^ To date, four transporters of lactate in the SLC16 family have been described,^13^ so we hypothesized that it should be possible to manipulate expression of all four paralog genes, to generate cell lines that are each dependent on individual transporters (Figure 1A). In this system, we expect cell line expressing only *SLC16A1* to be sensitive to SLC16A1 inhibitors, while not being affected by molecules selectively inhibiting SLC16A3, SLC16A7, or SLC16A8. Similarly, cell lines expressing only individual other paralog genes would be sensitive only to compounds specifically inhibiting the corresponding protein products, while compounds acting as dual inhibitors would affect cells lines expressing the cognate paralogs while having no effect on cell lines expressing other paralogs.

First, we compared the sensitivity of HAP1 WT, HAP1 *SLC16A1*^*−/−*^, and HAP1 *SLC16A3*^*−/−*^ cells to SLC16A1/SLC16A7 inhibitors AZD3965 and to BAY-8002 as well as the SLC16A3/SLC16A1 inhibitor Syrosingopine. We found that only *SLC16A3*^*−/−*^ cells were sensitive to AZD3965 and BAY-8002 (Figures 1B and S1B), while *SLC16A1*^*−/−*^ cells were more sensitive to Syrosingopine, in agreement with previously reported higher affinity of Syrosingopine to SLC16A3 compared to SLC16A1.^30^ To further confirm that this dependency is mediated by transport function, we re-expressed the transporters either as WT, or as a transport-deficient mutant, bearing a mutation in a conserved arginine residue critical for the function (SLC16A3^R278K^^32^ and SLC16A1^R313Q^^33^) and re-tested the inhibitors (Figures S1D and S1E). While WT transporters rescued the previously observed effect, the transport-deficient version did not, confirming that the synthetic lethality was dependent on the transport function (Figures 1C, 1D, and S1C). Interestingly, RNA-Seq showed that HAP1 cells endogenously do not express *SLC16A7* or *SLC16A8* (Figures 1E and S1F), further suggesting that fitness of HAP1 cells was dependent on expression of at least one functional lactate transporter. To create isogenic cell lines with dependencies on these two paralogs, we overexpressed (OE) individually *SLC16A7* and *SLC16A8* in HAP1 *SLC16A3*^*−/−*^ cell line and subsequently knocked-out *SLC16A1* (Figures 1A and S1G). No visible effect on cell fitness was observed upon the *SLC16A1* knockout, indicating that essential functions, and synthetic lethality, was rescued by exogenously introduced paralogs. Moreover, to test the dependency, we compared the sensitivity of these cells to AZD3965 and BAY-8002 and found that HAP1 *SLC16A3*^*−/−*^, *SLC16A1*^*−/−*^, *SLC16A7* OE were sensitive to AZD3965 and BAY-8002, while HAP1 *SLC16A3*^*−/−*^, *SLC16A1*^*−/−*^, *SLC16A8* OE cells were not (Figures 1F and S1H). These results are in agreement with previous studies reporting that both AZD3965 and BAY-8002 inhibit SLC16A7, albeit with lower affinity compared to SLC16A1,^16^^,^^29^ suggesting that these cell lines are dependent on the newly introduced SLC. These data showed that by manipulating expression of lactate transporters in HAP1 isogenic cell lines, survival/fitness dependency on individual paralogs can be generated.

### Chemical screening identified selective SLC16A3 inhibitors

**Figure 2:**
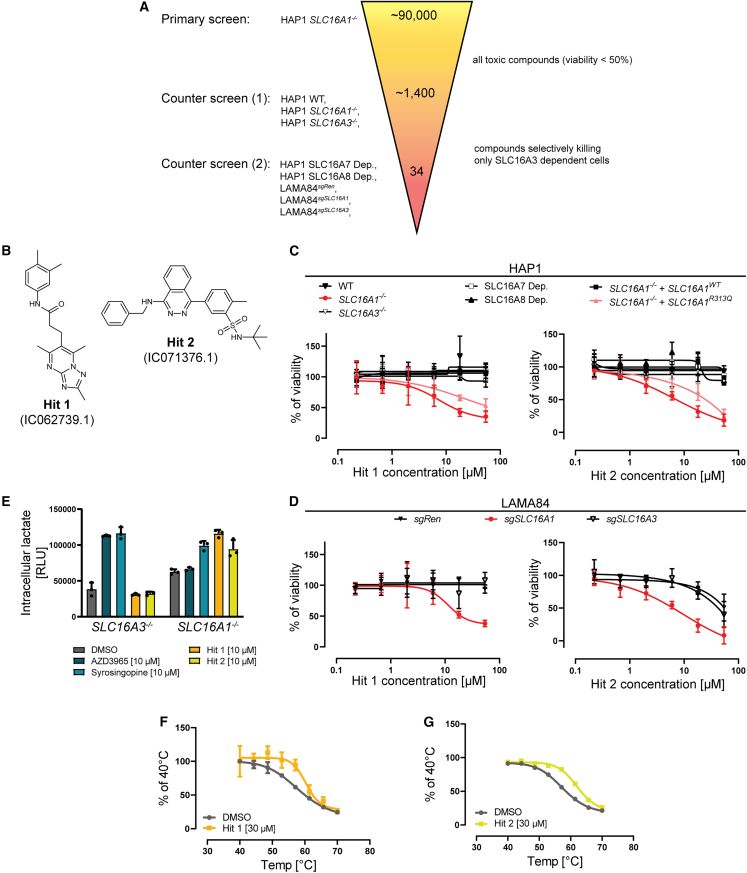
Chemical screening using PARADISO assay uncovered selective SLC16A3 (MCT4) inhibitors (A) Schematic representation of the SLC16A3 targeted chemical screening, indicating cell lines number of compounds, and hit criteria used in individual screening steps. (B) Chemical structures of representative hits based on triazolopyrimidine and phthalazine scaffolds. (C) Data from the chemical screen for HAP1 cells lines. (D) Data from the chemical screen for LAMA84 cell lines. (E) Effect of indicated molecules on intracellular lactate accumulation in HAP1 *SLC16A1*^*−/−*^ and HAP1 *SLC16A3*^*−/−*^ cell lines 6 h after treatment. (F) Thermal shift assay in lysates of HEK293T cells stably expressing *HiBiT-SLC16A3* upon treatment with Hit 1 or DMSO. Modified HiBiT lytic assay used as readout. (G) as in (F) but for Hit 2. All data are reported as mean ± SD.

Next, we used the PARADISO assay logic to convey a chemical screening campaign targeting SLC16A3 (Figure 2A). First, we used our in-house diversity-oriented library of approximately 90,000 compounds to screen for compounds reducing viability in HAP1 *SLC16A1*^*−/−*^ cells. We identified 1,410 compounds reducing the viability >50% compared to DMSO. To separate compounds acting on SLC16A3 from non-specific ones, we counter-screened the toxic compounds in dose response settings using not only HAP1 WT, but also *SLC16A1*^*−/−*^ and *SLC16A3*^*−/−*^ cell lines. This yielded 34 compounds able to selectively kill only the HAP1 *SLC16A1*^*−/−*^ cells, dependent on SLC16A3 function, but not any of the other two isogenic cell lines. To profile the selectivity further and to increase the confidence in our hits, we expanded the dose-ranging validations to additional cell lines including HAP1 with engineered SLC16A7 or SLC16A8 dependency, HAP1 *SLC16A1*^*−/−*^ overexpressing *SLC16A1*^*WT*^ or *SLC16A1*^*R313Q*^, and similarly to HAP1, we used LAMA84^sgRen^, LAMA84^sgSLC16A1^, and LAMA84^sgSLC16A3^ (SLC16A1-SLC16A3 synthetic lethality reported previously in,^31^ Figure S1I). We found seven compounds sharing a triazolopyrimidine scaffold and six compounds with shared phtalazine core (Table S1, examples in Figure 2B), which killed selectively only cell lines dependent on SLC16A3 (Figures 2C and 2D). Next, we confirmed that the treatment with compounds from both series resulted in intracellular lactate accumulation in HAP *SLC16A1*^*−/−*^, but not in HAP1 *SLC16A3*^*−/−*^ cells, suggesting that compounds inhibited SLC16A3 mediated lactate export (Figure 2E). To validate the binding of compounds to SLC16A3, we used the thermal shift assay using cell lysates and split nano-luciferase as readout. We observed a shift in the melting temperature of SLC16A3 upon treatment with both, triazolopyrimidine (Figure 2F) as well as phthalazine (Figure 2G) based compounds, suggesting binding to SLC16A3.

### slCeMM1 as a selective inhibitor of SLC16A3

**Figure 3:**
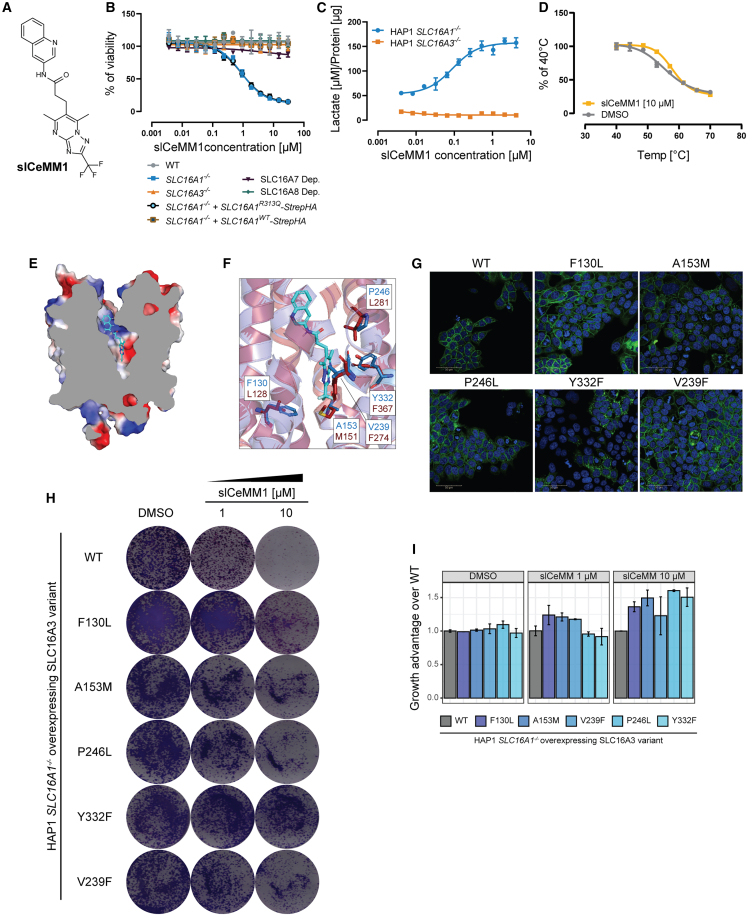
slCeMM1 is a selective and potent SLC16A3 (MCT4) inhibitor (A) Chemical structure of slCeMM1. (B) Viability of HAP1 cell lines upon treatment with slCeMM1 (SLC16A7 Dep. refers to HAP1 *SLC16A3*^*−/−*^ + *SLC16A7* OE + *sgSLC16A1* cells and SLC16A8 Dep. refers to HAP1 *SLC16A3*^*−/−*^ + *SLC16A8* OE + *sgSLC16A1* cells). (C) Effect of slCeMM1 treatment on intracellular lactate accumulation HAP1 *SLC16A1*^*−/−*^ or HAP1 *SLC16A3*^*−/−*^ after 6 h. (D) Thermal shift assay in lysates of HEK293T cells stably expressing *HiBiT-SLC16A3* upon treatment with Hit 1 or DMSO. (E) SLC16A3 homology model in outward-open conformation with docked slCeMM1. (F) Overlap of SLC16A3 homology model (blue) with docked slCeMM1 and CryoEM structure of SLC16A1 (red; PDB 6LYY). (G) Immunofluorescence staining of HiBiT-tag in HAP1 *SLC16A1*^*−/−*^ cells with overexpression of *SLC16A3* variants (all constructs expressed with HiBiT tag). Scale bars 50 μm. (H) Crystal violet staining of HAP1 *SLC16A1*^*−/−*^ cells, stably overexpressing indicated *SLC16A3* variants, treated with slCeMM1. (I) Growth advantage calculate from doubling times of HAP1 *SLC16A1*^*−/−*^ cells, stably overexpressing indicated *SLC16A3* variants, treated with slCeMM1. Data in (B-D) and (I) are presented as mean ± SD.

We further tested several analogs from both series using the PARADISO assay (Table S1). In addition, for a selection of compounds, the metabolic stability in liver microsomes was assessed (Figure S2A). These results led us to selection of slCeMM1 for further studies (Figure 3A). Further characterization showed that slCeMM1 inhibited growth of HAP1 cells dependent on SLC16A3 (IC_50_ = 0.88 μM), but not on SLC16A1, SLC16A7 or SLC16A8, and this effect was rescued by re-introduction of *SLC16A1*^*WT*^ but not transport-deficient mutant *SLC16A1*^*R313Q*^ (Figure 3B). The functional assay showed intracellular lactate accumulation upon treatment with slCeMM1 in SLC16A3 dependent cells (IC_50_ = 91 nM), but not in cells dependent on SLC16A1 (Figure 3C). Finally, binding to SLC16A3 was confirmed using the thermal shift assay (Figure 3D).

To investigate the mode of action of slCeMM1, as well as the possible structural basis for the selectivity toward SLC16A3, we built a homology model based on recently published structures of the paralog proteins^34^^,^^35^^,^^36^ and our structure-activity relationship (SAR) data. Docking of slCeMM1 suggested binding of the compound to the outward-open conformation of the transporter (Figure 3E). For the initial validation of the docking pose, we grouped residues important for the binding based on location in the protein structure (Figure S3A), mutated them to alanine and tested binding of slCeMM1 using the thermal shift assay. Loss of slCeMM1-mediated stabilization was observed in: (1) residues important exclusively for binding to the outward-open conformation (L68, F243, and P246; Figure S3B); (2) residues involved in lactate/H^+^ recognition and transport (K40, D274, and R278; Figure S3C); (3) residues localized in the central part of the structure (Y34, Y72, S156, Y332, and G336; Figure S3D). This suggested that at least a subset of residues in each group is critical for slCeMM1 binding. Comparing our model with structure of SLC16A1 bound to AZD3965^34^ suggested similarities between the binding sites (Figure S3E). Since our preliminary results showed that alanine mutant groups had reduced function (data not shown), we decided to focus on residues mediating selectivity between SLC16A3 and SLC16A1 using similar approach as Wang and colleagues.^34^ To do this, we selected several residues surrounding slCeMM1-binding pocket that are not conserved between SLC16A1 and SLC16A3 and mutated them individually for corresponding residues from SLC16A1 (F130L, A153M, V239F, P246L, and Y332F, Figure 3F). Immunofluorescence showed that staining pattern between the mutants and WT SLC16A3 is similar and that all mutants are capable of trafficking to the plasma membrane (Figure 3G). To determine the functionality, we overexpressed the mutants in HAP1 *SLC16A3*^*−/−*^ cells and tested the sensitivity to AZD3965. All mutants showed rescue phenotype comparable with WT SLC16A3, except V239F, which displayed weaker rescue (Figure S3F). Taken together, these data show that most of our mutants were functional; however, the function of V239F may be reduced. Next, we tested overexpression of SLC16A3 mutants on the effect of slCeMM1 in HAP1 *SLC16A1*^*−/−*^ cells. We observed that all tested mutations rescued the slCeMM1 effect partially (Figures 3H and 3I), indicating that tested residues are involved in slCeMM1 binding.

### slCeMM1-PAP uncovered selectivity at the proteome level

**Figure 4:**
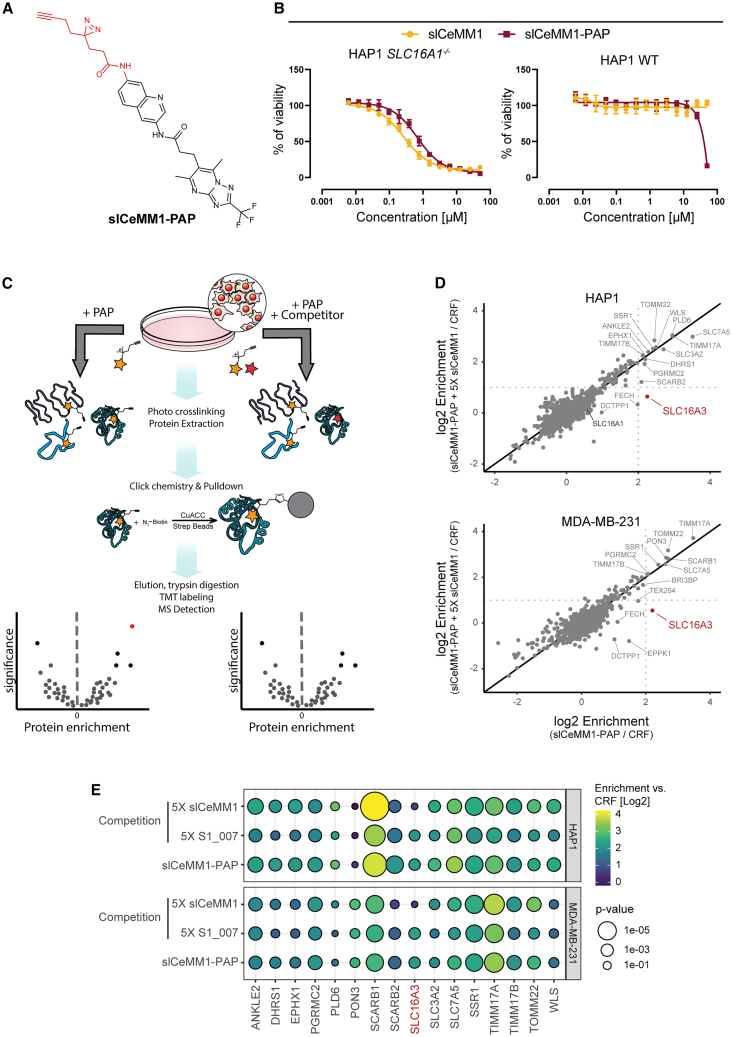
Chemoproteomic profiling of slCeMM1 selectivity (A) Chemical structure of slCeMM1-PAP. (B) Viability of HAP1 *SLC16A1*^*−/−*^ and HAP1 WT upon treatment with slCeMM1 or slCeMM1-PAP. Data reported as mean ± SD. (C) Schematic representation of the chemoproteomic experiment with slCeMM1-PAP. (D) Proteins enriched by 1 μM slCeMM1-PAP compared to enriched proteins in competition with 5 μM slCeMM1. Enrichment calculated over 50 μM CRF (structure corresponding to red part of slCeMM1-PAP in (A)). Dotted lines represent thresholds for protein enrichment (slCeMM1-PAP enrichment (log2) > 2) and competition (slCeMM1-PAP + 5X slCeMM1 enrichment (log2) < 1). (E) Comparison of abundances of proteins across tested conditions. Only proteins enriched by slCeMM1-PAP with Log2 enrichment >2 for at least one of the cell lines are shown. Color corresponds to Log2 enrichment over CRF and size corresponds to p value (ANOVA hypothesis test).

To determine the selectivity of slCeMM1 using an unbiased chemoproteomic approach, we synthesized a photoaffinity probe based on the slCeMM1 scaffold (slCeMM1-PAP; Figure 4A). The diazirine moiety enables covalent crosslinking upon photo activation and a terminal alkyne handle allows for conjugation to azide-reporter tags via click chemistry.^37^ First, we tested the biological activity of slCeMM1-PAP by comparing sensitivity of HAP1 *SLC16A1*^*−/−*^ and HAP1 WT with the parental compound. Both compounds showed similar effects, however the PAP was slightly less effective in *SLC16A1*^*−/−*^ cells and showed increased toxicity in WT cells at high concentrations (Figure 4B). This suggests that the compound is biologically active but may have off-target effects at high concentrations (Figure 4B).

We then assessed photoaffinity labeling of SLC16A3 in a HAP1 *SLC16A3-StrepHA* overexpressing cell line. Cells were treated with slCeMM1-PAP in the presence of different concentrations of slCeMM1 or its inactive, structurally related analog, S1_007 (Figure S2B, Table S1) as free competitors. The diazirine moiety of slCeMM1-PAP was then crosslinked with UV; cells were lysed followed by protein extraction and pulldown pre-enrichment of SLC16A3 using StrepHA tag. The alkyne handle of slCeMM1-PAP was then conjugated to the TAMRA-N_3_ reporter and visualized by SDS-PAGE and in-gel fluorescence scanning. The slCeMM1-PAP showed good labeling that was reduced by slCeMM1 competition, but not by S1_007 (Figure S2C).

To identify binding targets of slCeMM1, we performed quantitative mass-spectrometry (MS) based proteomics experiments using HAP1 and MDA-MB-231 cell lines. We compared the enrichment of proteins by slCeMM1-PAP and conditions where slCeMM1-PAP was competed with 5X concentration of slCeMM1 or S1_007 as control. Proteins that bind slCeMM1 should be enriched by slCeMM1-PAP, and their abundance should be reduced upon competition with slCeMM1, but not S1_007.^38^ To do this, we pre-treated cells with slCeMM1 or S1_007, followed by addition of slCeMM1-PAP, UV crosslinking, protein extraction, click-chemistry conjugation of a biotin-N_3_, pulldown enrichment, and MS analysis of interacting proteins (Figure 4C). In total, slCeMM1-PAP enriched 15 proteins in HAP1 and 9 proteins in MDA-MB-231 (log2 fold change over constant region fragment (CRF) > 2, Figures 4D and 4E). In both models, SLC16A3 showed the most prominent reduction in labeling upon competition with slCeMM1, suggesting overall good selectivity of the compound for this target. Moreover, the inactive structurally related compound S1_007 did not change the abundance of SLC16A3 (Figures S2D and 4E). A similar behavior was observed only in the case of the DCTPP1, however, this protein showed relatively low overall enrichment (slCeMM1-PAP/CRF log2 fold change of 0.98 in HAP1 and 1.02 in MDAMB231, respectively), suggesting that DCTPP1 could be a highly abundant off-target at high concentrations of slCeMM1. SLC16A1, endogenously expressed only in HAP1, was detected, but not enriched, supporting the paralog selectivity of slCeMM1. These data provide strong evidence for SLC16A3 engagement of slCeMM1. At the whole proteome level, slCeMM1 displays an overall good selectivity.

## Discussion

Development of highly specific cell-based assays for drug discovery targeting SLCs remains challenging mainly due to frequent functional redundancies. In this study, we established a strategy, the PARADISO assay that reduces redundancies, thus establishing an unambiguous SLC-phenotype relationship. We used the SLC16 family lactate transporters to establish a proof-of-concept. Previous studies focused on discoveries of inhibitors for SLC16A1/SLC16A3 relied on the use of cell lines with differential expression of lactate transporters. For example Quanz and colleagues used primarily DLD-1 to measure SLC16A1 and EVSA-T or 786-O for SLC16A3-mediated transport.^16^ Heinrich and colleagues used MDA-MB-231 for SLC16A3 and SNU-398 for SLC16A1.^20^ In our study we used a series of isogenic cell lines arranged in a logical cascade allowing the efficient identification of chemical agents acting in a highly selective manner in a cellular setting, in which the target is folded, modified, and localized naturally. Importantly, one of the strengths of the PARADISO system is that we could assess selectivity regarding the SLC16A7 and SLC16A8 proteins, products of gene paralogs that are usually not expressed individually in cell lines. Because of this counter-screens using cell lines with differential expression levels of SLC16 paralogs has been the only, suboptimal, option until now. This is particularly important because the closest related paralog of SLC16A3 is indeed SLC16A8.^15^ Additionally, the isogenic background of the PARADISO system provides confidence that differences in phenotype across the cell lines can be confidently attributed to the paralog that is assessed, rather than confounding dependencies arising from different genetic backgrounds. Using the PARADISO assay, we found two chemotypes selectively inhibiting SLC16A3 and we optimized the initial hits into slCeMM1. Furthermore, using an unbiased chemoproteomic approach we were able to confirm selectivity on a whole proteome level, suggesting further versatility of the PARADISO approach.

Several highly potent inhibitors of SLC16A3 were recently reported,^19^^,^^20^ and several less potent/selective SLC16A3 inhibitors were described previously.^30^^,^^39^^,^^40^^,^^41^ When finalizing this manuscript, Kawatkar and colleagues and Goldberg and colleagues reported another SLC16A3 inhibitors with nM potency, the latter based on a triazolopyrimidine scaffold.^22^^,^^23^ To the best of our judgment, slCeMM1 may well be the compound with the most rigorously defined selectivity among SLC16A3 paralogs, and with an experimentally assessed selectivity among cellular proteins, as defined by a proteomic approach. We therefore believe that our SLC16A3 inhibitor fulfills the criteria for a chemical probe^42^ and will thus become instrumental for the scientific community to assess the specific role of SLC16A3/MCT4 more broadly. Despite the possibly lower potency compared to the recently discovered SLC16A3 inhibitors,^19^^,^^20^^,^^22^^,^^23^ this puts slCeMM1 into a unique position for dissecting the specific biological role of SLC16A3 or validating it as a therapeutic target.^43^ In particular, slCeMM1 should be a valuable additional tool that helps to chemically validate SLC16A3 as a target based on experiments with gene knock-out/knock-down strategies. This includes cases where SLC16A3 has been suggested as possible target in disease areas such as cancer^44^^,^^45^^,^^46^^,^^47^^,^^48^ or rheumatoid arthritis.^49^ In addition, our study reports a homology model of SLC16A3 based on recently published structures as well as our SAR data. Our outward-open model complements the SLC16A3 model in the AlphaFold database, which is in inward-facing conformation, further expanding the possibilities for optimizations of drugs targeting SLC16A3.^50^

In summary, the presented study provides a framework for the development of highly specific cell-based assays for drug discovery, which may be widely applicable not only to SLCs, but also to other challenging targets possessing similar functional redundancies. In our view, the main limitation of this approach is the requirement for strong genotype-phenotype connections that may require a prior genetic screen for their identification. While genetic interactions solely among SLCs may be limited,^24^^,^^51^ an increasing number of studies are reporting unique SLC dependencies characterized by expression of metabolic genes. For example AML cells depend on myo-inositol import through SLC5A3 due to silencing of *ISYNA1*, an enzyme involved in myo-inositol biosynthesis,^52^ or ovarian cancer cells depend on phosphate exporter SLC53A1 (XPR1) due to overexpression of phosphate importer *SLC34A2*.^53^ In principle, the PARADISO assay design would also be applicable in these cases, expanding its potential to additional opportunities beyond SLC-SLC genetic interactions. In addition, using the PARADISO approach with cell viability as readout combines the advantages of target oriented and phenotypic screening, and thus is able to also capture compounds that modulate the target indirectly, yet in a highly specific manner.

### Limitations of the study

The PARADISO approach is not suitable, as designed, for genes that do not have paralogs or are essential to start with. Another limitation of our study is the sparse SAR information for the slCeMM1, as well as for the phthalazine series of compounds due to the limited access to medicinal chemistry. Similarly, while the liver microsome stability assay suggests overall good metabolic stability of slCeMM1, additional experiments to determine the *in vivo* usability of slCeMM1 are required. In addition, even though we were able to validate the homology model and binding of slCeMM1 with mutagenesis, our study implements a homology model instead of an experimentally determined structure.

## Significance

**The manuscript describes a chemical screening strategy allowing the straightforward identification of compounds acting selectively on an individual paralog among its peers. It is based on a cascade of assays using perfectly isogenic human cell lines engineered to be dependent on individual paralog genes. The approach is in principle applicable to any target expressed in a human cell line and thus has general validity. Here it is used for the identification and characterization of potent, specific, and selective inhibitors of the *SLC16A3* gene encoded membrane transporter of lactate and other monocarboxylates, an increasingly attractive target in a variety of diseases. Elucidation of the molecular basis for specificity compared to paralogs at the structural level and demonstration of selectivity among cellular proteins render the new compounds attractive chemical probes for the scientific community, empowering the precise assessment of its role in a variety of experimental settings. Several features make the two series of identified compounds attractive points of departure for drug discovery campaigns.**

## STAR★Methods

### Key resources table

**Table:** 

REAGENT or RESOURCE	SOURCE	IDENTIFIER
**Antibodies**		

Anti-HA High Affinity Rat mAb	Roche	Cat# 11867423001; RRID: AB_390918
Anti-pan ACTIN Rabbit polyclonal	Cytoskeleton, Inc.	Cat# AANO1; RRID: AB_10708070
MCT1 Antibody (H-1)	Santa Cruz Biotechnology	Cat# sc-365501; RRID: AB_10841766
Peroxidase-AffiniPure Goat anti-Rabbit IgG (H+L) antibody	Jackson ImmunoResearch Labs	Cat# 111-035-003; RRID: AB_2313567
Mouse Anti-HiBiT mAb (Clone 30E5)	Promega	Cat# CS2006A01; RRID: AB_2924793
Goat anti-Mouse IgG (H+L) Highly Cross-Absorbed Secondary Antibody, Alexa Fluor Plus 488	Thermo Fisher Scientific	Cat# A-11001; RRID: AB_2534069
Goat anti-Rat IgG (H+L) Cross-Adsorbed Secondary Antibody, Alexa Fluor 488	Thermo Fisher Scientific	Cat# A-11006; RRID: AB_2534074

**Bacterial and virus strains**		

One Shot™ Stbl3™ Chemically Competent E. coli	Invitrogen	Cat# C737303

**Chemicals, peptides, and recombinant proteins**		

AZD3965	MedChemExpress	Cat# HY-12750
BAY-8002	Selleckchem	Cat# S8747
Bortezomib	Selleckchem	Cat# S1013
Syrosingopine	Sigma-Aldrich	Cat# SML1908
slCeMM1	This study	N/A
slCeMM1-PAP	This study	N/A
S1_007	Chemspace	CSMS00334283126
Azide-PEG3-biotin	Sigma-Aldrich	Cat# 762024-25MG
5-TAMRA-Azide	Jena Bioscience	Cat# CLK-FA008-1
3-[3-(but-3-yn-1-yl)-3H-diazirin-3-yl]-N-methylpropanamide (CRF)	Enamine	Cat# EN300-7355163

**Critical commercial assays**		

CellTiter-Glo® Luminescent Cell Viability Assay	Promega	Cat# G7572
Nano-Glo® HiBiT Lytic Detection System	Promega	Cat# N3040
Lactate-Glo™ Assay	Promega	Cat# J5022
Q5® Site-Directed Mutagenesis Kit	New England Biolabs	Cat# E0554S

**Deposited data**		

HAP1 RNA sequencing data	RESOLUTE consortium	GEO: GSE234043
slCeMM1 Chemoproteomics	This study	https://doi.org/10.6019/PXD040089

**Experimental models: Cell lines**		

HAP1	Horizon Genomics	N/A
HAP1 SLC16A1-/-	Horizon Genomics	HZGHC002882c010
HAP1 SC16A3-/-	Horizon Genomics	HZGHC001844c001
MDA-MB-231	Gift from W. Berger’s lab	N/A
LAMA84 sgRen	Pemovska et al.^31^	N/A
LAMA84 sgSLC16A1	Pemovska et al.^31^	N/A
LAMA84 sgSLC16A3	Pemovska et al.^31^	N/A
HEK293T	ATCC	CRL-3216

**Oligonucleotides**		

*listed in the*Table S2		

**Recombinant DNA**		

pDONR221_SLC16A1	RESOLUTE consortium	Addgene #131934
pDONR221_SLC16A3	RESOLUTE consortium	Addgene #131899
pDONR221_SLC16A7	RESOLUTE consortium	Addgene #131947
pDONR221_SLC16A8	RESOLUTE consortium	Addgene #131959
pLentiCRISPRv2	Gift from F. Zhang lab	Addgene #52961
psPAX2	Gift from D. Trono lab	Addgene #12260
pMD2.G	Gift from D. Trono lab	Addgene #12259
pRRL	Bigenzahn et al.^54^	N/A
pRRL strepHA	Bigenzahn et al.^54^	N/A
pJTI R4 CMV-TO pA Vector	Thermo Fisher Scientific	Cat# A15006

**Software and algorithms**		

Prism v9	GraphPad Software	https://www.graphpad.com/scientific-software/prism/
R	The R Foundation	https://www.R-project.org/
Proteome Discoverer	Thermo Fisher Scientific	https://www.thermofisher.com/order/catalog/product/OPTON-31014

**Other**		

Pierce Streptavidin Agarose	Thermo Fisher Scientific	Cat# 20353
TMTpro 16plex Label Reagent Set	Thermo Fisher Scientific	Cat# A44521
StrepTactin Sepharose (50% suspension)	IBA	Cat# 2-1201-010

### Resource availability

#### Lead contact

Further information and requests for resources and reagents should be directed to and will be fulfilled by the lead contact, Giulio Superti-Furga (GSuperti@cemm.oeaw.ac.at).

#### Materials availability

Materials and reagents generated in this study are available upon a reasonable request from lead contact (GSuperti@cemm.oeaw.ac.at) and may require completed Material Transfer Agreement. Synthesis of slCeMM1 and slCeMM1-PAP is reported in the Synthesis section of methods.

### Experimental model and study participant details

#### Cell lines

All HAP1 (donor sex: male) cell lines were cultivated in IMDM medium. HEK293T (donor sex: female) were cultivated in DMEM medium. LAMA84 (donor sex: female) and MDA-MB-231 (donor sex: female) cell lines were cultivated in RPMI medium. All media were supplemented with 10% FBS (v/v) and P/S (100U/ml penicillin and 100 mg/ml streptomycin).

### Method details

#### Plasmids and stable cell line generation

pDONR221 vectors containing *SLC16A1*, *SLC16A3*, *SLC16A7* and *SLC16A8* cDNAs were gift from Resolute consortium. HiBiT tag and/or mutations were introduced using site directed mutagenesis (New England Biolabs; oligonucleotides listed in Table S2) and pDONR221 vectors. cDNAs were cloned into gateway-compatible vectors pRRL (for lentiviral expression, EF1a promotor driven expression, described previously^54^) or pJTI (for transient transfection, Thermo Fisher Scientific) using LR recombinase (Thermo Fisher Scientific). For CRISPR/Cas9 mediated knock-out generation, gRNA oligonucleotides were annealed and cloned into pLentiCRISPRv2 vector using golden gate assembly (SLC16A1 gRNA sequence: CACCGACAGACGTATAGTTGCTGTA). All vectors were confirmed by Sanger sequencing. Plasmids were chemically transformed into Stbl3 E.coli, plated on agar plates containing selection antibiotics and grew overnight. Single colonies were picked and further grow overnight in a liquid culture containing selection antibiotics. Plasmids were subsequently purified using QIAprep spin miniprep kit (Qiagen).

For the lentiviral transduction, HEK293T cells were transfected with psPAX2, pMDG2.G and expression vector using PEI. Approximately 12 hours post transfection, medium was replaced and after 48 – 72 hours the medium containing the lentiviral particles was harvested, filtered through 0.45 μm filter, mixed with Polybrene (5 μg/ml, Hexadimethrine bromide, Sigma Aldrich) and added to sub-confluent target cells. 24 hours after the transduction, medium was exchanged and 24 hours later selection antibiotics were added for 3 – 7 days to select infected cells. Knock-out and overexpression efficiency was evaluated by immunoblot and/or immunofluorescence.

#### Viability assay

Viability assays were run in 96 well plates, or 384 well plates, depending on number of tested conditions in experiment. Drugs were diluted in 2-fold or 3-fold serial dilution in complete growing medium to obtain 2X of final concentration (100 μl for 96 well plate, 25 μl for 384 well plate). Cells were then harvested, counted, diluted in complete growing medium and seeded in additional 100 μl per well for 96 well plate of 25 μl for 384 well plate respectively.

For 96 well plates, 2,500 cells per well were used and for 384 well plates, 1,000 cells per well were used respectively. Viability was measured after 72 hours using CellTiter-Glo assay (Promega) with a plate reader (SpectraMax i3x, Molecular Probes). Data were normalized to DMSO-treated controls (100% viability) and 5 μM Bortezomib controls (0% viability) and four-parameter dose response fitting curves were fitted using GraphPad Prism. All assays were run in at least technical triplicates and data is presented as mean +/- SD.

Testing of analogues of hits compounds was done first in 3-fold dilutions ranging from 30 μM to 4.6 nM. Compounds that were inactive were discarded after this step. Active compounds were re-tested in 2-fold dilutions with the concentration range adapted based on the first result.

#### Chemical screening

Prior to cell seeding, compounds were transferred into 384 well plates using acoustic liquid handler (Echo, Labcyte). Next, 1,000 cells (HAP1) or 1,500 cells (LAMA-84) per well were seeded using a dispenser (Thermo Fisher Scientific) in 50 μl of complete growth media per well. After 72 hours, viability was measured using CellTiter-Glo assay (Promega) with a plate reader (EnVision, PerkinElmer). Signal was then normalized to DMSO (100%) and Bortezomib (0%) controls.

In primary screen, 89,607 compounds were tested in concentration 10 μM (0.1% DMSO), in a single technical replicate using HAP1 *SLC16A1*^*-/-*^ cell line. For the counter screening only compounds reducing viability below 50% were progressed. During the first counter screening, concentrations 13, 4.3, 1.4 and 0.5 μM were tested in technical duplicate in HAP1 WT, HAP1 *SLC16A1*^*-/-*^ and HAP1 *SLC16A3*^*-/-*^ cell lines. Resulting dose response curves were inspected manually. For second round of counter screening, compounds selectively killing only HAP1 *SLC16A1*^*-/-*^ and structurally related compounds were selected. In this step compounds were tested in final concentrations 27, 9, 3, 1, 0.33 and 0.11 μM in technical triplicate in HAP1 WT, HAP1 *SLC16A1*^*-/-*^, HAP1 *SLC16A3*^*-/-*^, HAP1 SLC16A7 dependent, HAP1 SLC16A8 dependent, HAP1 *SLC16A1*^*-/-*^ + *SLC16A1*^*WT*^ OE, HAP1 *SLC16A1*^*-/-*^ + *SLC16A1*^*R313Q*^ OE, LAMA84^*sgRen*^, LAMA84^*sgSLC16A1*^ and LAMA84^*sgSLC16A3*^ cell lines.

#### Thermal shift assay in lysates

Cells were grown on 10 cm dishes until reaching confluency ∼80 – 90%. Cells were then washed with ice cold PBS and lysed with 3 ml of a lysis buffer (100 mM ammonium sulfate, 400 mM NaCl, 10% glycerol (v/v), 0.5% DDM (Sigma-Aldrich; w/v) and EDTA-free protease inhibitor cocktail (Roche) for 20 min on ice. Dishes were harvested by scraping, and lysates were cleared by centrifugation 14,000*g* for 20 min at 4°C. Supernatants were transferred to new tube, mix thoroughly, aliquoted and incubated with tested compounds for 60 min on ice. After that, individual aliquots were divided into PCR strips (50 μl per tube, 2 or 3 technical replicates per condition) and heated in gradient PCR thermocycler for 6 min (40°C to 70°C gradient), followed by 3 min incubation in 25°C. Next, samples were transfer on ice, technical replicates were pooled into 1.5 ml tubes, and protein aggregates were removed by centrifugation at 14,000*g* for 40 min at 4°C. For the detection of remaining protein, 25 μl of the supernatant was mixed with 5 μl of HiBiT detection mastermix (0.3 μl LgBIT Protein Nano-Glo + 0.6 μl HiBiT Lytic Substrate + 4.1 μl lysis buffer per sample) in black 384 well plate, incubated for 10 min at dark, and luminescence was measured on a plate reader (SpectraMax i3x, Molecular Probes).

All experiments were performed using cells expressing *SLC16A3* tagged with HiBiT. For experiment comparing SLC16A3 as a WT and as mutant groups, 293T cells were transiently transfected using PEI and pJTI constructs two days before the experiments. For thermal shift assays comparing DMSO and compounds, HAP1 cells with stable expression were used.

#### Intracellular lactate measurement

To assess the intracellular lactate accumulation, 30,000 cells per well were seeded in 96 well plate. Next day, cells were incubated with tested compounds for 6 hours at 37°C and 5% CO_2_ atmosphere, washed twice with 200 μl PBS, lysed with 22.5 μl 0.2 N HCl for 5 min while shaking, neutralized with 7.5 μl 1 M Tris-base buffer and mixed. Next, 30 μl of lactate detection reagent (Lactate-Glo, Promega) was added and plates were incubated for 60 min at RT and luminescence was measured with a plate reader (SpectraMax i3x, Molecular Probes). Absolute lactate concentrations were extrapolated from standard curve. To normalize the lactate levels to protein content, mirror plates were used. Here cells were lysed with RIPA buffer supplemented with Benzonase (15 min at room temperature (RT)) and protein concentration was measured with BCA assay (Thermo Fisher Scientific). All measurements were performed in technical triplicates, data are presented as mean +/- SD.

#### Chemoproteomics

##### Photoaffinity probe labelling of living cells

Cells were incubated with pre-treatment compounds (or DMSO) in serum free medium for 30 min in the incubator. Next slCeMM1-PAP (final concentration 1 μM) or CRF (final concentration 50 μM) were added, and cells were incubated for additional 5 min in the incubator. Cells were then irradiated with UV (365 nm, 5 min, 4°C), harvested, washed twice with ice cold PBS and cell pellets were stored at -80°C.

##### In-gel fluorescence analysis

Labelled pellets were lysed in buffer containing HEPES (50 mM, pH 8.0), NaCl (150 mM), NP-40 (0.5% v/v), PMSF (1 mM), EDTA-free protease inhibitor cocktail (Roche), Avidin (IBA, 2-0204-015; 1 μg/ml) and phosphatase inhibitor cocktail 2+3 (Sigma-Aldrich) for 15 min on RT. During the lysis samples were sonicated. Lysates were then cleared by centrifugation (14,000*g*, 20 min at 4°C). Protein concentration was measured with BCA assay (Thermo Fisher Scientific) and normalized across the samples. Streptactin Sepharose beads (IBA, 2-1201-010) were washed and equilibrated in the lysis buffer and incubated with the normalized samples for 1 hour at 4°C on a rotator (the amount of beads per sample corresponded to 40 μl of slurry). Next, samples were loaded to equilibrated Biospin columns (BioRad, 7326207) washed once with buffer containing HEPES (50 mM, pH 8.0), NaCl (150 mM), NP-40 (0.5% v/v), PMSF (1 mM) and EDTA-free protease inhibitor cocktail (Roche) and twice with buffer containing HEPES (50 mM, pH 8.0) and NaCl (150 mM). Captured protein was then eluted by incubating washed beads on thermoshaker (37°C, 300 rpm, 15 min) with 45 μl of PBS buffer containing 1% SDS, 2.5 mM D-biotin (Alfa Aeser, A14207), 2 mM MgCl_2_ and protease inhibitor cocktail. Eluates were then collected to separate tube by centrifugating the columns at 3,000*g* for 1 min. Volume of eluates was measured and normalized across the samples. Next, to facilitate copper catalyzed azide-alkyne cycloaddition (CuAAC) between the PAP and TAMRA reporter, 50 μl of the sample was mixed with 6 μl of freshly prepared master mix containing 25 μM 5-TAMRA-azide (Jena Bioscience, CLK-FA008), 0.1 mM Tris-[(1-benzyl-1H-1,2,3-triazol-4-yl)-methyl]-amin (TBTA, Sigma-Aldrich 678937-50MG), 1 mM CuSO_4_ (Sigma Aldrich, C1297-100G; freshly prepared) and 1 mM Tris(2-carboxyethyl)phosphine hydrochloride (TCEP; Sigma-Aldrich, 75259-1G), mixed and while protected from the light incubated at thermoshaker (25°C, 300 RPM) for 1 hour. Reaction was then stopped by adding 18.6 μl of 4X concentrated Laemmli sample buffer and 25 μl of samples were used for SDS-PAGE. After the separation, gels were de-stain (40% Methanol and 10% acetic acid) and fluorescent signal was visualized with BioRad ChemiDoc. As a loading control, gels were stained with Coomassie blue.

##### Sample preparation for MS chemoproteomic analysis

Labelled cell pellets were thaw on ice and resuspended in PBS buffer containing 1% SDS, 2 mM MgCl_2_, EDTA-free protease inhibitor cocktail (Roche) and Benzonase. Samples were incubated on thermoshaker (37°C, 300 rpm) for 30 min. Next, lysates were cleared by centrifugation (14,000*g*, 4°C, 30 min), supernatants were transferred to low protein binding tubes and protein was quantified with 660 nm Protein Assay Reagent (Thermo Fisher Scientific, 22660). Next, samples were normalized (based on the sample with the lowest protein concentration 545 μg per sample was used) and mixed with reagents to facilitate the CuAAC reaction (per sample: 450 μl of phosphate buffer; 25 μl of 5 mM Azide-PEG3-biotin (Sigma-Aldrich, 762024-25MG); 25 μl of 1:2 mixture of 20 mM CuSO_4_ (Sigma Aldrich, C1297-100G; freshly prepared) and 50 mM THPTA (Sigma Aldrich, 762342-500MG); 35 μl of 100 mM Aminoguanidine hydrochloride (Sigma Aldrich, 396494-25G); 35 μl of 100 mM L(+)-ascorbic acid sodium salt (Carl Roth, 3149.1; freshly prepared). Samples were then vortexed and incubated on a rotator for 1 hour at 25°C. Next, 3 ml of ice-cold acetone (Carl Roth, 7328.1) was added, samples were vortexed and left at -80°C for 30 to 60 min, to facilitate protein precipitation, followed by centrifugation (18,000*g*, 4°C, 30 min). Acetone was then carefully discarded, tubes with remaining protein pellets were left under fume hood to dry and stored at -20°C until samples were processed further.

Next, pellets were resuspended in 300 μl of 1% SDS by sonication (90 sec ON, 30 sec OFF, 5 cycles in total, using 4°C cooled Bioruptor Pico, Diagenode). Samples were then reduced by incubation of samples with 30 μl of 50 mM TCEP (Sigma-Aldrich, 75259-1G; final concentration 4.5 mM) for 1 hour at 56°C. After that, pH was adjusted by adding 80 μl HEPES (AppliChem, A6916,0125; 1 M, pH 7.5) and samples were alkylated by incubation with 45 μl of freshly prepared 200 mM iodoacetamide (Sigma Aldrich, I1149-5G; final concentration 20 mM) at 25°C for 30 min. Alkylated samples were then mixed with 100 μl streptavidin agarose beads (Thermo Scientific, 20353) washed and equilibrated with PBS. 1.35 ml PBS was added, and samples were incubated at 25°C for 1 hour on a rotator. Next, beads were separated from flow through by centrifugation, flow through was discarded, beads were resuspended in PBS buffer containing 0.2% SDS and transferred to equilibrated Minispin columns (Bio-Rad, 7326207). Beads were then washed 16-times with 0.5 ml PBS buffer containing 8 M Urea (Sigma-Aldrich, 51456-500G) and 4-times with 0.5 ml of PBS buffer. Columns were then closed with bottom caps, beads were resuspended in 1 ml digestion buffer (50 mM ammonium bicarbonate, 0.2 M guanidine hydrochloride and 1 mM CaCl_2_ in HPLC grade H_2_O (VWR, 83645.320P)), transferred to new low-protein binding tube, centrifuged for 30 sec, supernatant was discarded and beads were resuspended in 250 μl digestion buffer, 10 μl trypsin (Promega, V5117) was added (1 μg in total) and samples were left rotating at 37°C incubator overnight (∼14 hours).

Next day beads were separated by centrifugation (1,000*g*, 30 sec), supernatants were transferred to a new low-protein binding tube, beads were resuspended again with 200 μl of HPLC grade H_2_O, centrifuged and supernatants combined with the digest. For SPE clean-up, 200 μl tips were plunged with Empore C18 disk material (Sigma-Aldrich, 66883-U), 24 μl oligo R3 solution (Thermo Fisher Scientific, 1133903; 15 mg/ml in acetonitrile (ACN)) was added, tips were placed to collection tubes, centrifuged at 1,000*g* for 1 min, C18 was activated by washing twice with 100 μl 100% ACN (VWR, 83640.320) and subsequently equilibrated with 200 μl 0.1% trifluoroacetic acid (TFA; Sigma Aldrich, 1082620100). Samples were then acidified with 16 μl 30% TFA (∼1% final) and subsequently loaded on equilibrated tips, washed with 0.2% TFA and eluted by applying twice 50 μl of elution buffer (90% ACN + 0.01% TFA in H_2_O), each followed by centrifugation (1,000*g*, 3 min). Eluates were then dried in vacuum centrifuge (45°C, 1-2 hours until completely dry). Dried pellets were stored at -20°C until TMT-labelling.

For the TMTpro 16plex (Thermo Fisher Scientific, A44521) labelling, dried pellets were reconstituted in 15 μl HEPES (100 mM in HPLC grade H_2_O, pH 8.5). Respective TMTpro labels were added to the samples (4 μl of 0.01 mg/μl stocks dissolved in acetonitrile) and samples were vortexed and incubated at thermoshaker (25°C, 300 RPM) for 1 hour. Reaction was then stopped by adding 1.5 μl of freshly prepared 5% hydroxylamine solution in H_2_O for HPLC (Sigma Aldrich, 467804-10ML) and incubating samples on thermoshaker (25°C, 300 RPM) for 15 min. After that all samples were pooled into a single tube and 1 ml of freshly prepared ammonium formate ((AF; Sigma-Aldrich, 70221-25G-F), pH 10 adjusted with 25% ammonia solution) was added. Next, similarly as in previous step, 200 μl tip plunged with Empore C18 disk material and oligo R3 solution was prepared: tip was plunged with C18, 24 μl of oligo R3 solution was added, tip was placed to collection tubes, centrifuged at 1,000 x *g* for 1 min, C18 was activated by washing twice with 100 μl 100% ACN and washed again twice with 200 μl 20 mM AF solution. After that, pooled TMTpro labeled sample was applied to the tip in several steps, each followed by centrifugation and discarding of flow through. After whole sample volume was loaded on the tip, sample was washed with 200 μl 20 mM AF. Next elution was performed into 5 separate fractions, by on-tip high pH fractionation each with different ACN dilution in 20 mM FA: 1^st^ fraction with 16% ACN, 2^nd^ fraction with 20% ACN, 3^rd^ fraction with 24% ACN, 4^th^ fraction with 28% ACN, 5^th^ fraction with 80% ACN. Each elution step was done by applying 50 μl of appropriate elution solution, followed by centrifugation with 1,000*g* for 2 min and then applying additional 20 μl of appropriate elution buffer followed by 1,000*g* centrifugation for 1 min. All fractions were then dried in vacuum centrifuge set to 45°C. Dried pellets were then stored in -20°C until the acquisition, for which samples were reconstituted in 20 μl of 0.1 TFA.

##### 2D-RP/RP liquid chromatography – Tandem mass spectrometry analysis

Mass spectrometry analysis was performed on an Orbitrap Fusion Lumos Tribrid mass spectrometer (Thermo Fisher Scientific) coupled to a Dionex Ultimate 3000 RSLCnano system (Thermo Fisher Scientific) via a Nanospray Flex Ion Source (Thermo Fisher Scientific) interface. Peptides were loaded onto a trap column (PepMap 100 C18, 5 μm, 5 × 0.3 mm, Thermo Fisher Scientific) at a flow rate of 10 μL/min using 0.1% TFA as loading buffer. After loading, the trap column was switched in-line with an Acclaim PepMap nanoHPLC C18 analytical column (2.0 μm particle size, 75 μm IDx500mm, catalog number 164942, Thermo Fisher Scientific). The column temperature was maintained at 50°C. Mobile phase A consisted of 0.4% formic acid in water, and mobile phase B consisted of 0.4% formic acid in a mixture of 90% acetonitrile and 10% water. Separation was achieved using a four-step gradient over 151 min at a flow rate of 230 nL/min (increase of initial gradient from 6% to 9% solvent B within 1 min, 9% to 30% solvent B within 146 min, 30% to 65% solvent B within 8 min, 65% to 100% solvent B within of 1 minute and 100% solvent B for 6 minutes before equilibrating to 6% solvent B for 23 minutes prior to the next injection). In the liquid junction setup, electrospray ionization was enabled by applying a voltage of 1.8 kV directly to the liquid being sprayed, and non-coated silica emitter was used.

The mass spectrometer was operated in a data-dependent acquisition mode (DDA) and used a synchronous precursor selection (SPS) approach. For both MS2 and MS3 levels, we collected a 400–1600 m/z survey scan in the Orbitrap at 120 000 resolution (FTMS1), the AGC target was set to 'standard' and a maximum injection time (IT) of 50 ms was applied. Precursor ions were filtered by charge state (2-6), dynamic exclusion (60 s with a ±10 ppm window), and monoisotopic precursor selection. Precursor ions for data-dependent MSn (ddMSn) analysis were selected using 10 dependent scans (TopN approach). A charge-state filter was used to select precursors for data-dependent scanning. In ddMS2 analysis, spectra were obtained using one charge state per branch (from z=2 to z=5) in a dual-pressure linear ion trap (ITMS2). The quadrupole isolation window was set to 0.7 Da and the collision-induced dissociation (CID) fragmentation technique was used at a normalized collision energy of 35%. The normalized AGC target was set to 200% with a maximum IT of 35 ms. During the ddMS3 analyses, precursors were isolated using SPS waveform and different MS1 isolation windows (1.3 m/z for z=2, 1.2 m/z for z=3, 0.8 m/z for z= 4 and 0.7 m/z for z = 5). Target MS2 fragment ions were further fragmented by high-energy collision induced dissociation (HCD) followed by orbitrap analysis (FTMS3). The normalized HCD collision energy was set to 45% and the normalized AGC target was set to 300% with a maximum IT of 100 ms. The resolution was set to 50 000 with a defined scanning range of 100 to 500 m/z. Xcalibur Version 4.3.73.11 and Tune 3.4.3072.18 were used to operate the instrument.

#### Immunofluorescence staining and imaging

Cells were seeded onto poly-L-lysine hydrobromide (Sigma-Aldrich, P6282) coated black 96 well plates (PerkinElmer). Upon reaching sub-confluency, cells were fixed with formaldehyde (4% in PBS) for 15 min on RT, permeabilized in blocking solution (0.3% Triton X-100, 10% FBS in PBS) for 1 hour at RT. Staining with primary antibodies was performed overnight at 4°C in blocking solution, cells were subsequently washed with blocking buffer (3x) and fluorophore conjugated secondary antibody diluted in blocking buffer was applied for 1 hour at RT. Next, cells were washed with blocking buffer (3x), stained with DAPI (1:1,000 in PBS) for 15 min at RT and imaged using Opera Phenix High Content Screening System (PerkinElmer). A full list of used antibodies is described in key resources table.

#### Crystal violet proliferation assay

For the proliferation assay, 5,000 cells per well were seeded into 6 well plate and treated immediately. Medium with drugs was refreshed every 3 days. After 7 days, cells were washed with PBS, fixed (4% formaldehyde in PBS) for 15 min at RT, washed with water and stained with crystal violet (0.1% in water). Plates were then washed again with water and left to dry.

#### Doubling-time assay

15,000 cells per well were seeded and treated immediately in 6 well plates. Cells were harvested and counted with CASY counter (Roche) after 1, 2 and 3 days. The doubling time was estimated using exponential growth equation in Graphpad Prism, and the growth advantage was calculated by comparing the doubling time of WT and variants in each condition. All experiments were performed in technical duplicates.

#### qPCR

RNA was isolated using RNeasy Mini kit (Qiagen). Genomic DNA was digested using DNase I (Fermentas, #EN0525) and the cDNA was synthesized using RevertAid reverse transcriptase (Thermo Fisher Scientific, EP0441) and oligo dT primers according to manufacturer’s protocols. qPCR was performed on LightCycler 480 (Roche) using Luna Universal qPCR Master Mix (New England Biolabs, M3003L) according to manufacturer’s protocol. Expression changes were calculated using ΔΔCt method. All samples were run in biological triplicate and technical duplicates.

#### Immunoblotting

Cells for immunoblotting were grown to sub-confluency in 6 well plates, washed with ice-cold PBS, harvested by scrapping, washed again and pellets were stored at -80°C until further processing. Next, pellets were thaw and lysed with RIPA buffer (25 mM Tris/HCl pH 7.6, 150 mM NaCl, 1% NP-40, 1% sodium deoxycholate, 0.1% SDS, EDTA-free protease inhibitor cocktail (Roche) and phosphatase inhibitors 2+3 (Sigma-Aldrich) for 15 min on ice. Lysates were cleared by centrifugation (14,000*g*, 20 min, 4°C), protein concentration was determined with BCA assay (Thermo Fisher Scientific) and protein levels were normalized across all samples. Laemmli sample buffer was added to protein extracts without boiling. Samples were resolved with SDS-PAGE and transferred to nitrocellulose membranes Amesham Protran 0.45 μm (GE Healthcare). Membranes were stained with ponceau red, washed, blocked in 5% milk for 1 hour at RT and incubated in primary antibodies overnight at 4°C. Next day, membranes were washed (3x, 5 min) and incubated with horseradish peroxidase conjugated secondary antibodies for 1 hour at RT. Membranes were then washed (3x 5 min), and signal was detected using ECL Western blotting system (Thermo Fisher Scientific) on BioRad ChemiDoc.

#### Homology modeling and docking

Homology models of SLC16A3 in inward-open and outward-open conformations were built. First, the most reliable templates to generate models were identified using the HHPred server,^55^ as well as visually inspecting available structures of SLC16A3 close homologs, i.e. structures of SLC16A1 (PDB IDs: 6LZ0, 6LYY, 7CKR, 7KO^34^), SLC16A7 (PDB ID: 7BP3^35^) and sfMCT (PDB ID: 6HCL^36^). The best templates were prioritized based on their sequence similarity to the target, their conformational state, the resolution of their structures, as well as whether or not they were bound to a ligand. SLC16A1 was selected as the most suitable template for homology modeling, as it presents a high sequence similarity to SLC16A3 (49%) and has been experimentally determined in multiple conformational states bound to substrates and inhibitors.^34^

A sequence alignment of all the 14 members of the human SLC16 family was generated using PROMALS 3D.^56^ For each conformation (outward-open, using 6LYY as template and inward-open, using 7CKO as template) of SLC16A3, 100 models were generated using MODELLER 9.22.^57^ Models were ranked according to their Z-DOPE score.^58^ The quality of the best scoring models were further evaluated with PROCHECK^59^: the outward-open and inward-open models presented 96.2 and 97.4 % of residues in the most favored regions of the Ramachandran plot, respectively.

Induced-Fit docking calculations were performed with the Schrödinger suite.^60^ The models were first prepared using the Protein Preparation Wizard^61^ within Maestro with default options. During this step, the proteins are protonated and energy-minimized. The docked ligands were prepared with LigPrep, with default options, with the exception of the pH range set at 7±0.5.

The binding sites were defined around the centroid ligand bound to the structure used as template for homology modelling (i.e. AZD3965 in the outward-facing state, and 7ACC2 in the inward-facing state).

Induced fit docking of slCeMM1 was performed using OPLS3e force field, a ligand conformational sampling within 2.5 kcal/mol, a van der Waals scaling of 0.5, 20 maximum number of poses, Prime refinement of residues within 5 Å of ligand poses. Interaction fingerprint clustering was then performed on the resulting poses. Finally, the best-scored poses within the most populated cluster were selected for visual inspection.

#### Liver microsome stability assay

Metabolic stability in human, mouse and rat liver microsomes was tested by WuXi DMPK Nanjing as follow:

Working solutions of tested compounds were prepared by diluting 5 μl of compound (10 mM DMSO stock) with 495 μl acetonitrile (ACN). NADPH cofactors were prepared by diluting β-Nicotinamide adenine dinucleotide phosphate reduced form, tetrasodium salt (NADPH·4Na (Vendor: BONTAC, Cat. No. BT04) into 10 mM MgCl_2_ to obtain 10 mM working solution concentration. Liver microsomes were prepared by dilution of Human (Corning, Cat No. 452117, Lot No. 38297), CD-1 Mouse (Xenotech, Cat no. M1000, Lot no. 2110092) or SD Rat (Xenotech Cat No. R1000, Lot No. 2110178) in 100 mM potassium phosphate buffer to final concentration 0.56 mg/ml. As a stop solution was used cold ACN (4°C) containing 200 ng/ml tolbutamide and 200 ng/ml labetalol as internal standards.

Blank plate was prepared by combining 54 μl liver microsomes with 6 μl of NADPH cofactor, followed by addition of 180 μl quenching solution. Incubation plates (T60 and NCF60 (no cofactor)) were prepared by combining 445 μl of microsome working solution with 5 μl of compound working solution in pre-warmed plate, followed by mixing. For the NCF60 sample, 50 μl of buffer was added and plate was incubated for 60 min at 37°C while shaking. Quenching plate T0 was prepared by mixing 180 μl of quenching solution and 6 μl of NADPH cofactor followed by chilling to prevent evaporation. 54 μl of the mixture from T60 plate was transferred to quenching plate, followed by addition of 44 μl NADPH to T60 incubation plate and 60 min incubation at 37°C while shaking. At 5, 15, 30, 45 and 60 min, 180 μl quenching solution was added to quenching plate, mixed, and 60 μl sample was serially transfer from T60 plate per time point to quenching plate. For NCF60, 60 μl sample was transferred from NCF60 incubation plate to quenching plate containing quenching solution at 60 min time point. Next to tested compounds, Testosterone, Diclofenac and Propafenone was measured as controls. Final concentration of each component in the incubation medium was 0.5 mg protein/ml for microsomes, 1 μM tested compound, 0.99% ACN, 0.01% DMSO.

All sampling plates were shaken for 10 min, centrifuged at 4,000 rpm for 20 min at 4C, 80 μl supernatant transferred to 240 μl HPLC water, mixed and shaken for 10 min. Each bioanalysis plate was sealed and shaken for 10 min prior to LC-MS/MS analysis.

#### Synthesis of slCeMM1 and slCeMM1-PAP

##### slCeMM1

**Figure:**
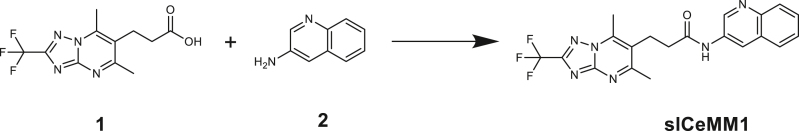

To a stirred solution of compound 1 (2 g, 6.94 mmol, 1 eq) in DCM (20 mL), 1,1'-Carbonyldiimidazole (1.46 g, 9.03 mmol, 1.3 eq) was added. The reaction mixture was stirred at RT for 1 h. Then, compound 2 (1 g, 6.94 mmol, 1 eq) was added, and the resulting solution was stirred at RT overnight. After that, the reaction mixture was washed with H_2_O (20 mL x 2). The organic layer was separated, dried over anhydrous Na_2_SO_4_, filtered, and evaporated under reduced pressure to afford 2.5 g of crude target material, which was purified by FC (CHCl_3_:CH_3_CN) to obtain pure product slCeMM1 (1 g, 35% yield).

1H NMR (400 MHz, dmso) δ 10.43 (s, 1H), 8.87 (d, J = 2.5 Hz, 1H), 8.70 (d, J = 2.5 Hz, 1H), 7.93 (t, J = 7.4, 7.4 Hz, 2H), 7.69 – 7.60 (m, 1H), 7.57 (t, J = 7.5, 7.5 Hz, 1H), 3.19 (t, J = 7.8, 7.8 Hz, 2H), 2.85 (s, 3H), 2.76 (s, 3H), 2.72 (dd, J = 8.8, 6.9 Hz, 2H).

LC-MS m/z 415.0 [M+1]

##### slCeMM1-PAP

**Figure:**
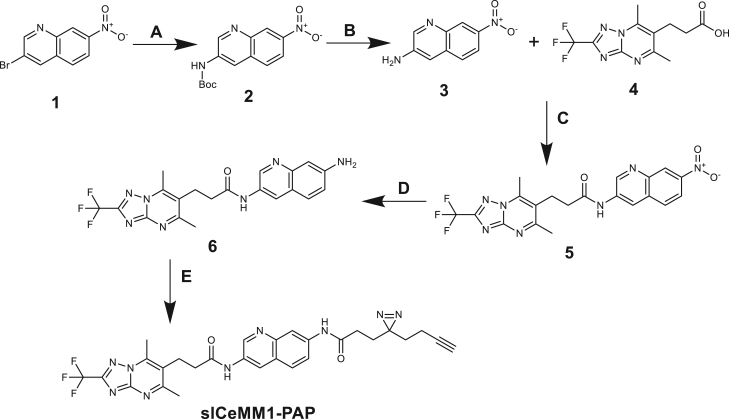

**Step A**: 3-bromo-7-nitroquinoline (compound **1**) (2.81 g, 11.1 mmol), Cs_2_CO_3_ (10.85 g, 33.31 mmol), Pd_2_(dba)_3_ (0.25 g, 0.27 mmol), Xantphos (0.32 g, 0.55 mmol), tert-butyl carbamate (1.43 g, 12.21 mmol) and dioxane (40 mL) were placed into the Schlenk flask (100 mL). The resulting solution was stirred at 100°C for 16 h. After completion of the reaction, the mixture was filtered through SiO_2_, the pad was washed with EtOAc (100 mL), and the combined filtrates were evaporated in a vacuo to obtain tert-butyl (7-nitroquinolin-3-yl) carbamate (compound **2**) (1.53 g, 47.7 % yield).

**Step B**: To a stirred solution of tert-butyl (7-nitroquinolin-3-yl) carbamate (1.53 g, 5.29 mmol) in MeOH (10 mL), HCl (4 M solution in dioxane, 20 mL) was added. The resulting solution was stirred at RT for 16 h. The solvent was evaporated to obtain 7-nitroquinolin-3-aminium chloride (compound **3**) (1.01 g, 85% yield) as a brown solid.

**Step C**: 7-nitroquinolin-3-aminium chloride (compound **3**) (1.01 g, 4.5 mmol) was dissolved in DMF (10 mL), and 3-(5,7-dimethyl-2-(trifluoromethyl)-[1,2,4]triazolo[1,5-a]pyrimidin-6-yl)propanoic acid (compound **4**) (1.18 g, 4.09 mmol), HATU (2.02 g, 5.31 mmol) were added thereto followed by addition of N, N-Diisopropylethylamine (2.11 g, 16.36 mmol, 2.85 mL). The resulting mixture was stirred at 50 °C for 16 h. The solvent was evaporated in a vacuum. EtOAc was added to the residue, and a brown precipitate was observed. The insoluble solids were collected by filtration and washed with EtOAc. After drying under vacuum, the desired product 3-(5,7-dimethyl-2-(trifluoromethyl)-[1,2,4] triazolo [1,5-a]pyrimidin-6-yl)-N-(7-nitroquinolin-3-yl)propenamide (compound **5**) was obtained as a brown powder (1.26 g, 61.1% yield).

**Step D**: 3-(5,7-dimethyl-2-(trifluoromethyl)- [1,2,4] triazolo [1,5-a]pyrimidin-6-yl)-N-(7-nitroquinolin-3-yl) propenamide (compound **5**) (1.26 g, 2.74 mmol) was dissolved in AcOH (5 mL), and 10% Pd/C (0.3 g) was added. The flask was evacuated, backfilled with hydrogen gas from a balloon, and left to stir overnight at RT. The reaction mixture was filtered, and the solvent was rotary evaporated to obtain N-(7-aminoquinolin-3-yl)-3-(5,7-dimethyl-2-(trifluoromethyl)- [1,2,4] triazolo [1,5-a] pyrimidin-6-yl) propenamide (compound **6**) (1 g, 84.67% yield).

**Step E**: N-(7-aminoquinolin-3-yl)-3-(5,7-dimethyl-2 -(trifluoromethyl)- [1,2,4] triazolo[1,5-a] pyrimidin-6-yl)propenamide (compound **6**) (1 g, 2.32 mmol) was dissolved in DMF (7 mL), and 3-(3-(but-3-yn-1-yl)-3H-diazirin-3-yl)propanoic acid (0.38 g, 2.32 mmol), HATU (1.14 g, 3.01 mmol) were added thereto followed by addition of N, N-Diisopropylethylamine (1.2 g, 9.28 mmol, 1.62 mL). The resulting mixture was stirred at RT for 48 h. The solvent was evaporated to obtain a crude product. After HPLC (Сolumn: SunFire 100∗19 mm, 5 μM, Mobile Phase: system 0.5-6.5 min 63% water- 37% ACN 30 ml/min (loading pump 4 ml ACN) target mass 578) was obtained 3-(3-(but-3-yn-1-yl)-3H-diazirin-3-yl)-N-(3-(3-(5,7-dimethyl-2-(trifluoromethyl)-[1,2,4]triazolo[1,5-a] pyrimidin-6-yl)propanamido)quinolin-7-yl)propenamide (**slCeMM1-PAP**) (0.239 g, 17.8% yield).

1H NMR (400 MHz, dmso) δ 10.38 (s, 1H), 10.22 (s, 1H), 8.79 (d, J = 2.5 Hz, 1H), 8.60 (d, J = 2.5 Hz, 1H), 8.33 (d, J = 1.9 Hz, 1H), 7.85 (d, J = 8.9 Hz, 1H), 7.64 (dd, J = 8.8, 2.1 Hz, 1H), 3.18 (t, J = 7.9, 7.9 Hz, 2H), 2.90 – 2.81 (m, 4H), 2.76 (s, 3H), 2.72 – 2.67 (m, 2H), 2.21 (t, J = 7.6, 7.6 Hz, 2H), 2.03 (td, J = 7.5, 7.4, 2.7 Hz, 2H), 1.80 (t, J = 7.6, 7.6 Hz, 2H), 1.63 (t, J = 7.4, 7.4 Hz, 2H).

LC-MS m/z 578.4 [M+1]

### Quantification and statistical analysis

Statistical parameters are reported in the figure legend of the paper.

#### Chemoproteomics

Following data acquisition, the acquired raw data files were processed using the Proteome Discoverer v.2.4.1.15 platform, with a TMT16plex quantification method selected. In the processing step, we used the Sequest HT database search engine and the Percolator validation software node to remove false positives with a false discovery rate (FDR) of 1% at the peptide and protein level under stringent conditions. The search was performed with full tryptic digestion against the human proteome (Canonical, reviewed, 20 304 sequences) and appended known contaminants and streptavidin, with a maximum of two allowable miscleavage sites. Methionine oxidation (+15.994 Da) and protein N-terminal acetylation (+42.011 Da), as well as methionine loss (-131.040 Da) and protein N-terminal acetylation with methionine loss (-89.030 Da) were set as variable modifications, while carbamidomethylation (+57.021 Da) of cysteine residues and tandem mass tag (TMT) 16-plex labeling of peptide N termini and lysine residues (+304.207 Da) were set as fixed modifications. Data were searched with mass tolerances of ±10 ppm and ±0.6 Da for the precursor and fragment ions, respectively. Results were filtered to include peptide spectrum matches with Sequest HT cross-correlation factor (Xcorr) scores of ≥1 and high peptide confidence assigned by Percolator. MS3 signal-to-noise (S/N) values of TMTpro reporter ions were used to calculate peptide/protein abundance values. Peptide spectrum matches with precursor isolation interference values of ≥70%, SPS mass matches ≤65%, and average TMTpro reporter ion S/N ≤10 were excluded from quantification. Both unique and razor peptides were used for TMT quantification. Correction of isotopic impurities was applied. Data were normalized to total peptide abundance to correct for experimental bias and scaled “to all average”. Protein ratios are directly calculated from the grouped protein abundances using an ANOVA hypothesis test. The mass spectrometry proteomics data have been deposited to the ProteomeXchange Consortium via the PRIDE^62^ partner repository with the dataset identifier PXD040089 and 10.6019/PXD040089.

#### Liver microsome stability assay

The equation of first order kinetics was used to calculate T_1/2_ and Cl_int(mic)_(μl/min/mg):

$$\begin{array}{c} C_{t}=C_{0}\cdot e^{-k_{e}\cdot t}\\ when\;C_{t}=\frac{1}{2}C_{0}\text{,}\\ T_{\frac{1}{2}}=\frac{\mathrm{Ln}\;2}{k_{e}}=\frac{0.693}{k_{e}}\\ {CL}_{\mathrm{int}\left(mic\right)}=\frac{0.693}{In\;vitro\;T_{\frac{1}{2}}}\cdot \frac{1}{\frac{mg}{ml}microsomalprotein\;in\;reaction} \end{array}$$

## Data Availability

• All data reported in this paper will be shared by the lead contact upon request. The mass spectrometry proteomics data have been deposited to the ProteomeXchange Consortium via the PRIDE database (http://www.proteomexchange.org) and are publicly available as of the date of publication. Accession numbers are listed in the key resources table. RNA seq data have been deposited at GEO and are publicly available as of the date of publication. Accession numbers are listed in the key resources table. • This paper does not report original code. • Any additional information required to reanalyze the data reported in this paper is available from the lead contact upon request.

## Supplemental information

Document S1. Figures S1–S3

Table S1. SAR data. Data for hits from the chemical screening, and for analogs of hits (including vendors and catalog numbers for commercially available compounds), related to figure 2

Table S2. Oligonucleotide list. List of olignocleotides used in this study, related to STAR methods

Document S2. Article plus supplemental information
