# Supplementary material for: Paralog-dependent isogenic cell assay cascade generates highly selective SLC16A3 inhibitors
Source: Cell Chem Biol. 2023 Aug 17;30(8):953–964.e9. doi: 10.1016/j.chembiol.2023.06.029 (PMC10437005; doi:10.1016/j.chembiol.2023.06.029)
Supplement: Document S1. Figures S1–S3 [file mmc1.pdf]

Cell Chemical Biology, Volume *30*

Supplemental information

Paralog-dependent isogenic cell assay cascade generates highly selective SLC16A3 inhibitors

Vojtech Dvorak, Andrea Casiraghi, Claire Colas, Anna Koren, Tatjana Tomek, Fabian Offensperger, Andrea Rukavina, Gary Tin, Elisa Hahn, Sarah Dobner, Fabian Frommelt, Andras Boeszoermenyi, Viktoriia Bernada, J. Thomas Hannich, Gerhard F. Ecker, Georg E. Winter, Stefan Kubicek, and Giulio Superti-Furga

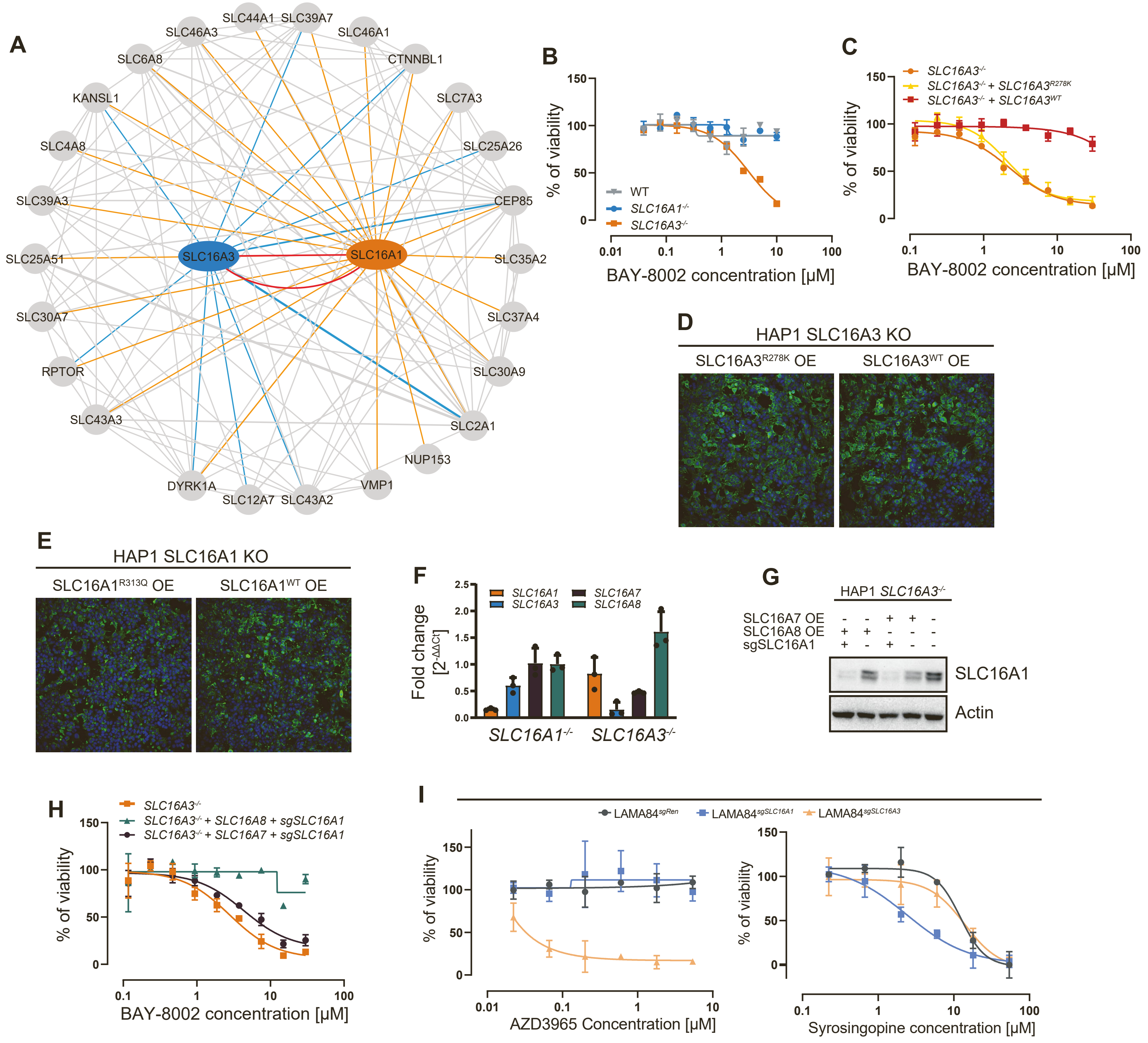

Supplementary Figure 1: Synthetic lethality between *SLC16A1* (MCT1) and *SLC16A3* (MCT4) can be explored chemically, related to Figure 1.

(A) Genetic interaction network of *SLC16A1* and *SLC16A3* (1st degree neighborhood, adapted from Girargi et al.[1], negative interactions with GI score < -5 shown).

(B) Sensitivity of HAP1 WT, HAP1 *SLC16A1*$^{-/-}$ and HAP1 *SLC16A3*$^{-/-}$ cell lines to BAY-8002 (SLC16A1/SLC16A7 inhibitor).

(C) Sensitivity of HAP1 *SLC16A3*$^{-/-}$, HAP1 *SLC16A3*$^{-/-}$ with *SLC16A3*^{WT} or HAP1 *SLC16A3*$^{-/-}$ with *SLC16A3*^{R278K} overexpression to BAY-8002.

(D) Immunofluorescence staining of HA-tag in HAP1 *SLC16A3*$^{-/-}$ with *SLC16A3*^{WT} or *SLC16A3*^{R278K} (both constructs expressed with StrepHA tag).

(E) Same as (D) but for with *SLC16A1*^{WT} or *SLC16A1*^{R313Q} in HAP1 *SLC16A1*$^{-/-}$ cells.

(F) qPCR showing relative change in expression of lactate transporters in HAP1 *SLC16A1*$^{-/-}$ and HAP1 *SLC16A3*$^{-/-}$ cell lines compared to WT.

(G) Western blotting confirmation of SLC16A1 protein level redustion in SLC16A7 (HAP1 *SLC16A3*$^{-/-}$ + *SLC16A7* OE + *sgSLC16A1*) and SLC16A8 (HAP1 *SLC16A3*$^{-/-}$ + *SLC16A8* OE + *sgSLC16A1*) dependent cell.

(H) Sensitivity of SLC16A7 and SLC16A8 dependent cells to BAY-8002.

(I) Sensitivity of LAMA84^{sgRen}, LAMA84^{sgSLC16A1} and LAMA84^{sgSLC16A3} to AZD3965 and Syrosingopine.

Data presented as mean +/- SD.

A

Compound	Human Liver Microsomes		Rat or Mouse Liver Microsomes	
	$T_{1/2}$ [min]	$CL_{int(mic)}$ (µl/min/mg)	$T_{1/2}$ [min]	$CL_{int(mic)}$ (µl/min/mg)
**slCeMM1**	**>145**	**<9.6**	**84.8**	**16.3**
S1_002	49.1	28.3	4.7	296.3
S1_005	9.9	139.9	3.5	309
S1_003	6.4	215.5	3.8	366.7
S1_015	2.2	591.6	1.6	881.5
S2_002	46.2	30	37	37.4
S2_003	8.2	169.3	2.2	627
S2_004	2.3	599.3	0.7	1899.2
S2_005	0.6	2159.7	0.6	2474

B

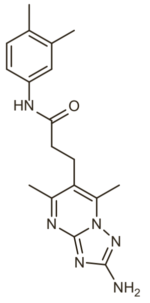

S1_007

C

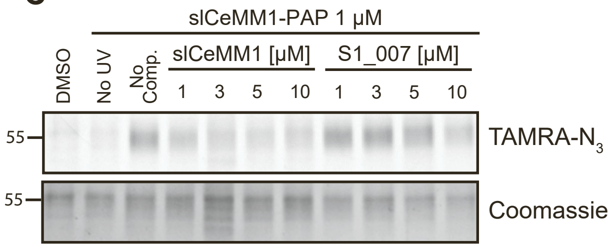

D

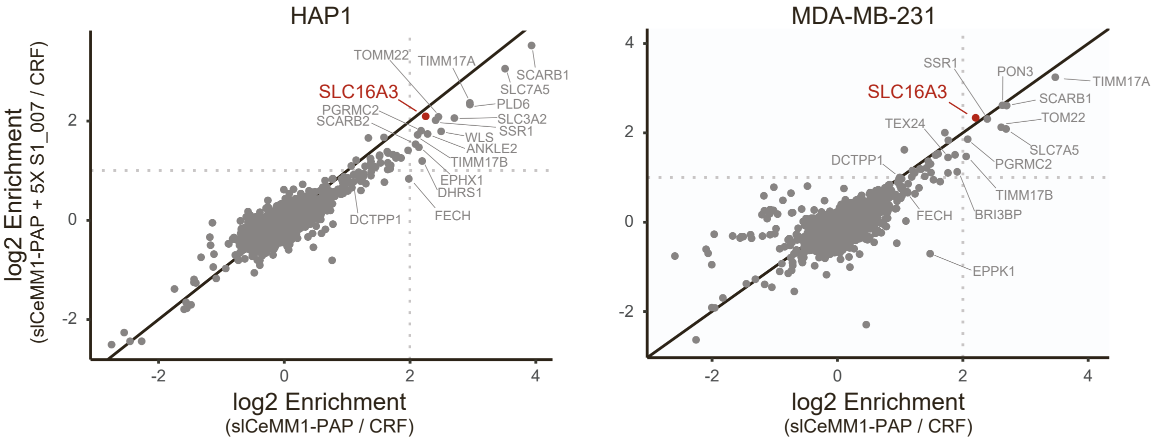

Supplementary Figure 2: Additional slCeMM1 characteristics (related to Figure 3) and slCeMM1-PAP chemoproteomics (related to Figure 4).

(A) Liver microsome stability assay data summary. slCeMM1 and S2_003 tested in mouse liver microsomes, rest of the compounds tested in liver microsomes from rat.

(B) Chemical structure of S1_007.

(C) In-gel fluorescence of SLC16A3-StrepHA pulldown enriched samples. Coomassie staining shown as a loading control.

(D) Proteins enriched by 1 µM slCeMM1-PAP compared to enriched proteins in competition with 5 µM S1_007. Enrichment calculated over 50 µM CRF (structure corresponding to red part of slCeMM1-PAP in Fig. 4A). Dotted lines represent thresholds for protein enrichment (slCeMM1-PAP enrichmenr (log2) > 2) and competition (slCeMM1-PAP + 5X S1_007 enrichment (log2) < 1).

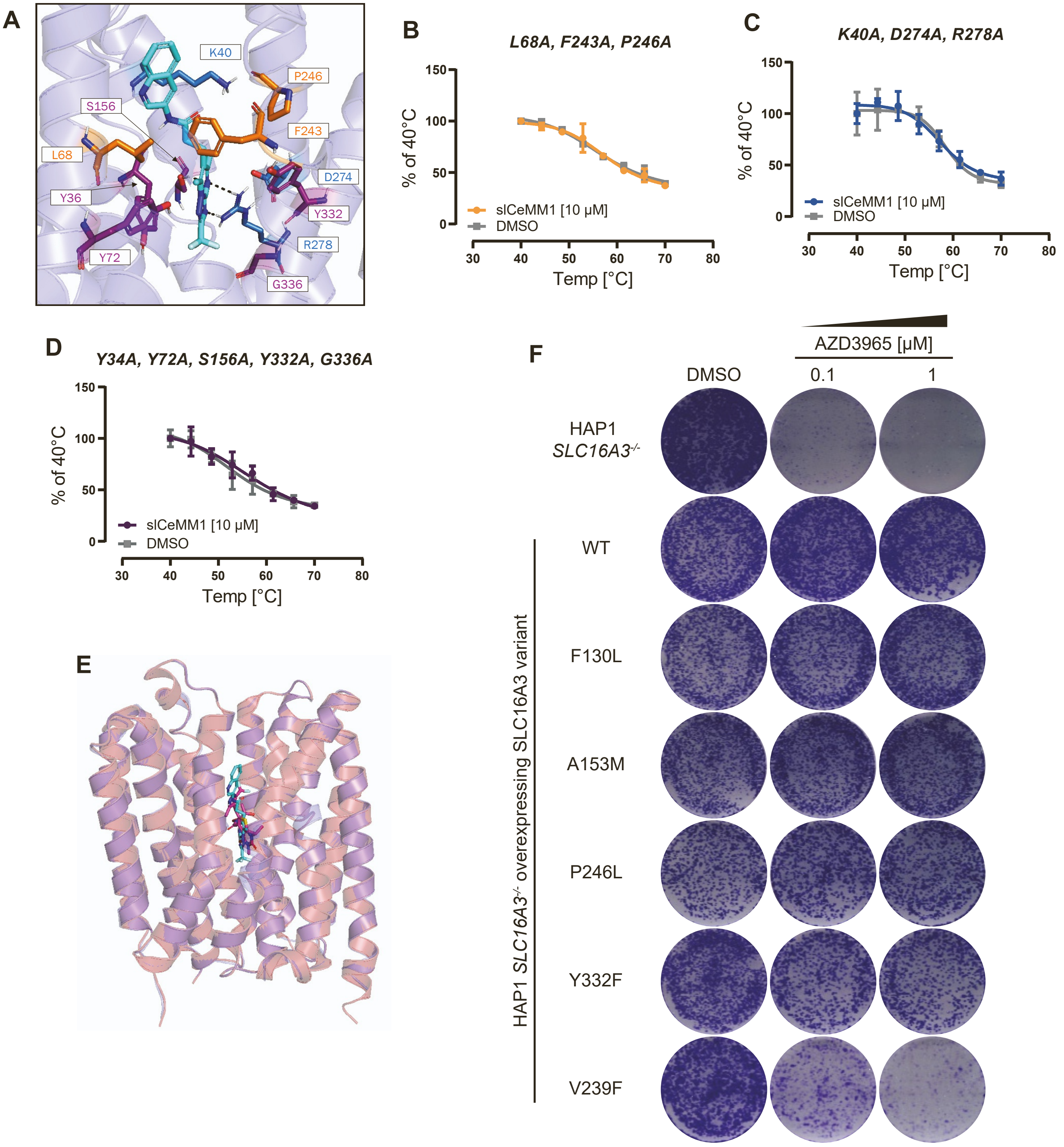

Supplementary Figure 3: Additional SLC16A3 (MCT4) homology model data, related to Figure 3.
(A) SLC16A3 homology model in outward-open conformation with docked slCeMM1, highlighted residues involved in slCeMM1 binding, color coded based on position in the structure (yellow - "outward group", purple - "central group") or function (blue - residues involved in lactate/H^+ recognition).
(B) Thermal shift assay in lysates of HEK293T transiently transfected to express *HiBiT-SLC16A3* with mutations in residues involved in slCeMM1 binding, localized close to extracellular opening of the transporter cavity ("outward group" - L68A, F243A, P246A).
(C) Same as in (B), but with mutated residues involved in lactate/H^+ recognition ("functional group" - K40A, D274A, R278A).
(D) Same as in (B), but with mutated residues localized closer to the center of the structure ("central group" - Y34A, Y72A, S156A, Y332A, G336A).
(E) Overlap of SLC16A3 homology model (blue) with docked slCeMM1 (cyan) and CryoEM structure of AZD3965 (magenta) bound to SLC16A1 (red)[2].
(F) Crystal violet staining of HAP1 *SLC16A3*$^{-/-}$ cells, stably overexpressing indicated SLC16A3 variants, treated with slCeMM1.

Supplementary References

1. Girardi, E., Fiume, G., Goldmann, U., Sin, C., Müller, F., Sedlyarov, V., Srndic, I., Agerer, B., Kartnig, F., Meixner, E., et al. (2020). A systematic genetic interaction map of human solute carriers assigns a role to SLC25A51/MCART1 in mitochondrial NAD uptake. bioRxiv, 2020.08.31.275818.

2. Wang, N., Jiang, X., Zhang, S., Zhu, A., Yuan, Y., Xu, H., Lei, J., and Yan, C. (2021). Structural basis of human monocarboxylate transporter 1 inhibition by anti-cancer drug candidates. Cell 184, 370-383.e13. 10.1016/j.cell.2020.11.043.
